# Sparse-view reconstruction and noise reduction in ^99m^Tc-prostate specific membrane antigen prostate imaging

**DOI:** 10.1097/MNM.0000000000002079

**Published:** 2025-11-11

**Authors:** Sayna Jamaati, Masoumeh Dorri Giv, Xiaotong Hong, Ramin Sadeghi, Amin Saber Tanha, Masoud Noroozi, Hossein Arabi

**Affiliations:** a Department of Energy Engineering, Sharif University of Technology, Tehran; b Nuclear Medicine Research Center, Department of Nuclear Medicine, Ghaem Hospital, Medical University of Mashhad, Mashhad, Iran; c Department of Biomedical Engineering, Southern Medical University, Guangzhou, China; d Department of Biomedical Engineering, Faculty of Engineering, University of Isfahan, Isfahan, Iran; e Department of Nuclear Medicine & Molecular Imaging, Geneva University Hospital, Geneva, Switzerland

**Keywords:** prostate cancer, prostate specific membrane antigen, single photon emission computed tomography, sparse-view reconstruction

## Abstract

**Purpose:**

This preliminary study evaluates the feasibility and clinical value of sparse-view acquisition in ^99m^Tc-prostate specific membrane antigen (PSMA) single photon emission computed tomography (SPECT) prostate imaging. This study also compares the performance of traditional Gaussian filtering vs. an edge-preserving nonlocal means (NLM) filter in the sparse-view SPECT approach.

**Methods:**

Ten patients with biopsy-proven prostate cancer (Gleason 4–10) underwent full-angle acquisitions (60 views), which were decimated to 30, 15, and 10 views. Images were reconstructed with ordered-subsets expectation maximization (9 iterations, 5 subsets) and postfiltered with a Gaussian kernel or NLM. Quantitative performance metrics included mean absolute percentage error (MAPE), normalized root mean square error (NRMSE), peak signal-to-noise ratio (PSNR), and normalized bias (NB). Two nuclear medicine physicians (10–12 years’ experience) provided blinded visual assessments.

**Results:**

Halving the views to 30 preserved diagnostic accuracy (MAPE_ROI1 ≤ 7%). Reducing to 15 views introduced sampling artifacts consistent with violation of the angular Nyquist limit, and 10 views produced unacceptable artifacts (MAPE_ROI1 > 50%). For 30-view reconstructions, NLM outperformed Gaussian, improving NRMSE by up to 39.3%, PSNR by 4.5%, and NB by 18.55%. Expert readers confirmed these trends, with high interobserver agreement (intraclass correlation coefficient = 0.83) for 30 views.

**Conclusion:**

Sparse-view ^99m^Tc-PSMA SPECT with 30 views appears feasible for routine and emergency prostate imaging in this setting. Reconstructions from 15 views showed aliasing from angular undersampling and are not recommended without anti-aliasing strategies and further validation. This work characterizes the added value of NLM postreconstruction filtering in PSMA-SPECT and motivates larger studies.

## Introduction

Single photon emission computed tomography (SPECT) generates a three-dimensional map of gamma-ray emissions from radionuclides. This imaging technique provides valuable functional data on organs and tissues, allowing for the detection of abnormalities at a functional level before anatomical changes become apparent. SPECT is widely used to assess normal physiological processes and diagnose a range of conditions, including cardiovascular diseases, neurological disorders, thyroid dysfunction, and cancer [1–7]. However, scan durations could take 20–30 min or longer, depending on the area being imaged and the clinical protocol. For example, whole-body scans or more detailed protocols can push the time closer to an hour [8,9]. This extended scan time presents several disadvantages, including patient discomfort and increased likelihood of patient movement during the procedure. These factors can compromise image quality, necessitate repeated scans, and lead to diagnostic delays [10,11].

Sparse-view scanning is a technique in tomography modalities to mitigate their high dose and long scan times. Significantly developed for computed tomography scanners, it has gained remarkable attention for its ability to substantially reduce factors like lower scanning time by decreasing the number of views [12].

It is important to recognize that limited angular sampling has been employed in specific SPECT applications, notably in cardiac imaging, since the early days of SPECT technology. For example, early cardiac SPECT studies noted that limited-angle acquisition could shorten scan time. Go *et al*. [13] reported that 180 ° sampling reduced acquisition time and improved contrast, although they concluded it was less reliable than full 360 ° acquisition. Tamaki *et al*. [14] demonstrated that limited-geometry imaging (seven-pinhole tomography) could yield clinically useful myocardial tomographic images, establishing the feasibility of non-full-angle approaches in cardiac SPECT even if their focus was on image quality rather than deliberate time reduction. Despite this historical context, the current study broadens the application of sparse-view scanning to include noncardiac imaging to investigate the advantages of reducing acquired data on time reduction and comfort of the patients in other fields of SPECT imaging. Conventional SPECT systems rely on the acquisition of a large number of angular views to reconstruct high-quality diagnostic images, which often results in prolonged scan times. These lengthy imaging sessions can be challenging for patients, especially those who struggle to remain still for extended periods. In contrast, sparse-view SPECT significantly reduces the number of angular projections required during a scan [10,15,16].

One of the most notable advantages of this method is its ability to alleviate patient discomfort. Long scan times in conventional SPECT systems can be physically and emotionally taxing for patients, particularly for those with mobility limitations, chronic pain, or anxiety. By reducing the number of views collected, sparse-view SPECT allows for quicker imaging sessions, minimizing the time patients spend in an uncomfortable scanning environment. Additionally, shorter scans help decrease the likelihood of motion-related artifacts, which can occur when patients move during the procedure, thereby enhancing the quality and reliability of the diagnostic images [11,16].

However, one of the significant challenges associated with the sparse-view method is the increased susceptibility to noise and artifacts in the reconstructed images. The reduced number of angular views inherently limits the amount of data available for image reconstruction, which can lead to incomplete or inaccurate representations of the scanned region. To address these challenges, it becomes crucial to employ advanced reconstruction algorithms and appropriate filtering techniques that are specifically designed to mitigate the noise and suppress artifacts. These methods are essential for enhancing image quality and ensuring that the diagnostic reliability of sparse-view SPECT is maintained, despite the reduced data acquisition [11,15–17].

Iterative reconstruction (IR) is a prominent technique for reconstructing sparse-view projection data. IR methods are well-grounded in mathematical theory and effectively employ projective geometry and physical principles during the data acquisition process [6,18].

The maximum likelihood expectation maximization (MLEM) algorithm, a widely recognized statistical iterative reconstruction method, seeks to generate an estimate that maximizes the likelihood function, thereby providing the best fit to the observed data. Despite being a conventional IR approach, the MLEM algorithm is known for its slow convergence rate. In response, the ordered subset expectation maximization (OSEM) algorithm has been introduced as a notable alternative, offering a significantly faster convergence rate [19,20]. However, despite improvements in the reconstruction part, sparse-view SPECT images are often affected by noise, which can degrade image quality and compromise diagnostic accuracy.

A widely used method for minimizing noise in tomography images is applying postreconstruction filters, such as the Gaussian filter. However, these denoising techniques can introduce quantitative inaccuracies, lead to image quality degradation, and create artifacts [21–24]. To mitigate these challenges, edge-preserving noise reduction techniques like nonlocal means (NLM) filters have been developed, aiming to retain critical features and edges of images while effectively reducing noise, thereby improving image quality in SPECT [25–27].

In this study, the ^99m^Tc-PSMA (prostate specific membrane antigen) SPECT images of patients were collected. The diagnostic efficacy of ^99m^Tc-PSMA SPECT for prostate cancer provides a comprehensive evaluation of this imaging modality’s effectiveness in detecting prostate cancer.

A significant advantage of ^99m^Tc-PSMA SPECT is its accessibility and cost-effectiveness compared to ^68^Ga-PSMA PET. The widespread availability of technetium-99 m generators facilitates broader clinical application, especially in regions with limited access to PET imaging facilities. This increased accessibility can lead to earlier detection and treatment of prostate cancer, potentially improving patient outcomes [28].

To date, most sparse-view studies have focused on other fields of SPECT; very few studies have addressed ^99m^Tc-PSMA SPECT. This study fills this gap by evaluating sparse-view ^99m^Tc-PSMA imaging with the robust OSEM algorithm and by comparing Gaussian filtering against an edge-preserving NLM approach.

## Method

### Ordered subset expectation maximization-nonlocal means algorithm

The measured projection data ${\left\{y_{i}\right\}}_{i}^{M}$ in SPECT are modeled as independent Poisson counts with means $p_{i}$, given by


$$\begin{array}{l} p_{i}=\underset{j=1}{\overset{J}{\sum }}\;a_{ij}x_{j}, \end{array}$$ (1)

where $p_{i}$ is the expected count in the projection bin i, $a_{ij}$ is the element of the system matrix representing the probability that an emission from pixel j is detected in bin i, $x_{j}$ is the (unknown) expected emission rate from pixel j [19,29]. The likelihood of observing $y_{i}$ given x is:


$$\begin{array}{l} P\left(y_{i}|x\right)=\frac{p_{i}^{y_{i}}}{y_{i}}e^{-p_{i}} \end{array}$$ (2)

and the total log-likelihood is


$$\begin{array}{l} \ell \left(x\right)=\underset{i=1}{\overset{M}{\sum }}\;(y_{i}\ln p_{i}-p_{i}) \end{array}$$ (3)

To maximize ℓ(x) under the constraint $x_{j}\geq 0$, the OSEM algorithm is employed [19,29]. Partition the set of projection bins into B disjoint subsets $S_{1},\ldots .,,S_{B}$. Denote by $x_{j}^{\left(n,b\right)}$ the estimate of pixel j after processing subset $,S_{B}$ in the n-th iteration, with


$$\begin{array}{l} x_{j}^{\left(n,,0\right)}=x_{j}^{\left(n,-1B\right)} \end{array}$$

Define the forward projection of the current estimate as


$$\begin{array}{l} {\hat{p}}^{\left(n,b-1\right)}=\underset{j=1}{\overset{J}{\sum }}\;a_{ik}x_{k}^{\left(n,b-1\right)} \end{array}$$ (4)

where ${\hat{p}}^{\left(n,b-1\right)}$ is the predicted count in the b-th subset based on the current estimate. Then for each subset b=1,…, B the update is


$$\begin{array}{l} x_{j}^{\left(n,b\right)}x_{j}^{\left(n,b-1\right)}\times \frac{\underset{i\in S_{b}}{\sum }a_{ij}\frac{y_{i}}{{\hat{p}}_{i}^{\left(n,b-1\right)}}}{\underset{i\in S_{b}}{\sum }a_{ij}} \end{array}$$ (5)

where the numerator of the fraction measures how well the actual counts $y_{i}$ agree with the predicted values ${\hat{p}}_{i}$, and the denominator of the fraction $\underset{i\in S_{b}}{\sum }\;a_{ij}$ normalizes over each subset, in accordance with the original OSEM formulation. After b=B, it is set


$$\begin{array}{l} x_{j}^{\left(n+1,0\right)}x_{j}^{\left(n,B\right)} \end{array}$$ (6)

To optimize the reconstructed image, postreconstruction filters are applied to enhance the results. The NLM method is used for optimizing and filtering images; it is an effective denoising algorithm that reduces noise by averaging pixels with similar intensity patterns, regardless of their spatial proximity [26]. This method preserves image details, such as edges and textures, by considering the entire image for noise reduction, rather than just neighboring pixels, and it can be defined as follows:


$$\begin{array}{l} x\left(\;j\right)=\underset{i\in N_{j}}{\sum }\;w(\;j,i)x\left(i\right) \end{array}$$ (7)

In this context, *x(i*) refers to the pixel intensity of *i* in the initial image before employing the filter, while *N*_*j*_ defines the local search area. The term *x(j*) indicates the estimated intensity at pixel *j* in the final image after employing the filter, and *w(j,i*) is computed based on the degree of similarity between pixels *j* and *i* [26]. The formula of *w(j,i*) is as follows:


$$\begin{array}{l} w(j,i)=\frac{1}{Z(j)}exp\left\{\frac{-∥x(\nu_{j})-x(\nu_{i})∥{}_{2,\alpha }^{2}}{h^{2}}\right\} \end{array}$$ (8)

In this context, *z(j*) is a normalization factor that ensures the sum of all weights equals 1. *x(v*_*j*_) and *x(v*_*i*_) represent vectors of pixel values within the local search window. Two similar windows are denoted by *v*_*j*_ and *v*_*i*_. The squared *l*_*2*_ norm is represented by $._{2}^{2},,,$ without applying any additional weighting to the norm, and the α parameter acts as the SD of the Gaussian kernel, determining the rate at which similarity between patches decreases as the squared Euclidean distance increases. The parameter *h* is the filter parameter and indicates the level of noise in the image.

### Data acquisition

This scan was performed using a ^99m^Tc-PSMA radiopharmaceutical, and images were taken using SPECT methods. At first, 20 to 25 mCi of the radiopharmaceutical was injected into the patient through a peripheral vein, and about 3 to 4 h after the injection, the radiopharmaceutical was absorbed in the prostate area, and 1–2 SPECT images were taken focusing on the pelvic region, and kidney, and especially the amount of radiopharmaceutical uptake in the prostate gland. All the study cases were diagnosed with prostate cancer from pathology with a Gleason score between 4 and 10. In this study, projections from ten different patients with various Gleason scores were used. ^99m^Tc-PSMA SPECT was acquired on a GE Discovery 670 Pro over 360 ° with 60 projections (6 ° steps) and a 128 × 128 matrix; images were reconstructed to 128 × 128 × 128 with 4.43 mm³ voxels. The acquisition time per view was fixed (~17.5–20 s/view), giving a total of 17.5–20 min for 60 views. Reduced-view sets (30, 15, 10 views; with 12 °, 24 °, 36 ° interval for each reduced projection respectively) were generated by uniform decimation of the 60-view sinograms with unchanged per-view counts/noise; thus, effective total counts and scan time scale linearly with number of views (≈8.8–10.0, 4.4–5.0, 2.9–3.3 min, for 30, 15, 10 projections approximately). Reconstruction used OSEM (nine iterations, five subsets), followed by the filters detailed in Methods.

### Evaluation

To quantitatively evaluate the reconstruction results, mean absolute percentage error (MAPE), peak signal-to-noise ratio (PSNR), normalized root mean square error (NRMSE), and normalized bias (NB) metrics were employed. The total views (60 views) images were regarded as reference images, and reduced-view images (30, 15, and 10 views) were regarded as reduced, since the number of total projections has reduced to fewer numbers. The equations of four metrics are defined as follows:


$$\begin{array}{l} MAPE=\left|\frac{\mu^{ref}-\mu^{reduced}}{\mu^{ref}}\right|\times 100\% \end{array}$$ (9)

where $\mu^{ref}$ represents the mean intensity values in the corresponding region of interest (ROI) in the reference image, and $\mu^{reduced}$ denotes the mean intensity values in the corresponding ROI in the reduced images.


$$\begin{array}{l} NRMSE=\sqrt{\frac{\overset{N}{\underset{J=1}{\sum }}{\left({x^{reduced}}_{J}-{x^{ref}}_{J}\right)}^{2}}{\overset{N}{\underset{J=1}{\sum }}{\left({x^{ref}}_{J}-\mu \right)}^{2}}} \end{array}$$ (10)

where $x^{reduced}$ and $x^{ref}$ denote the pixel value or each pixel *J* in the reduced and reference images, respectively, while μ represents the average values of pixels in the reference image. *N* refers to the total number of pixels.


$$\begin{array}{l} PSNR=10\times \log\left(\frac{{\left(max\left(x^{reduced}\right)\right)}^{2}}{x^{reduced}-{x^{ref}}^{2}}\right) \end{array}$$ (11)

where $x^{reduced}$ represents the reduced image, and $x^{ref}$ refers to the reference image.


$$\begin{array}{l} NB=\frac{\mu^{ref}-\mu^{reduced}}{var\left(\mu^{ref}\right)} \end{array}$$ (12)

where $\mu^{ref}$ is the mean intensity value in the ROI1 of the reference image, $\mu^{reduced}$ is the mean intensity value in the ROI1 of the reduced image, and $var\left(\mu^{ref}\right)$ is the variance of the reference image in the ROI1. NB criterion has only been considered in ROI1 to investigate the maintenance of signal in the high uptake region.

In addition to the quantitative evaluation, a qualitative assessment was also conducted by two physicians (with 10–12 years of experience) to confirm the clinical relevance and significance of this study. The physicians rated the images based on Table 1.

**Table 1:** Likert scales were used in the reader study

Score	Image quality
1	Very poor
2	Poor
3	Fair
4	Good
5	Excellent

## Results

**Fig. 1:**
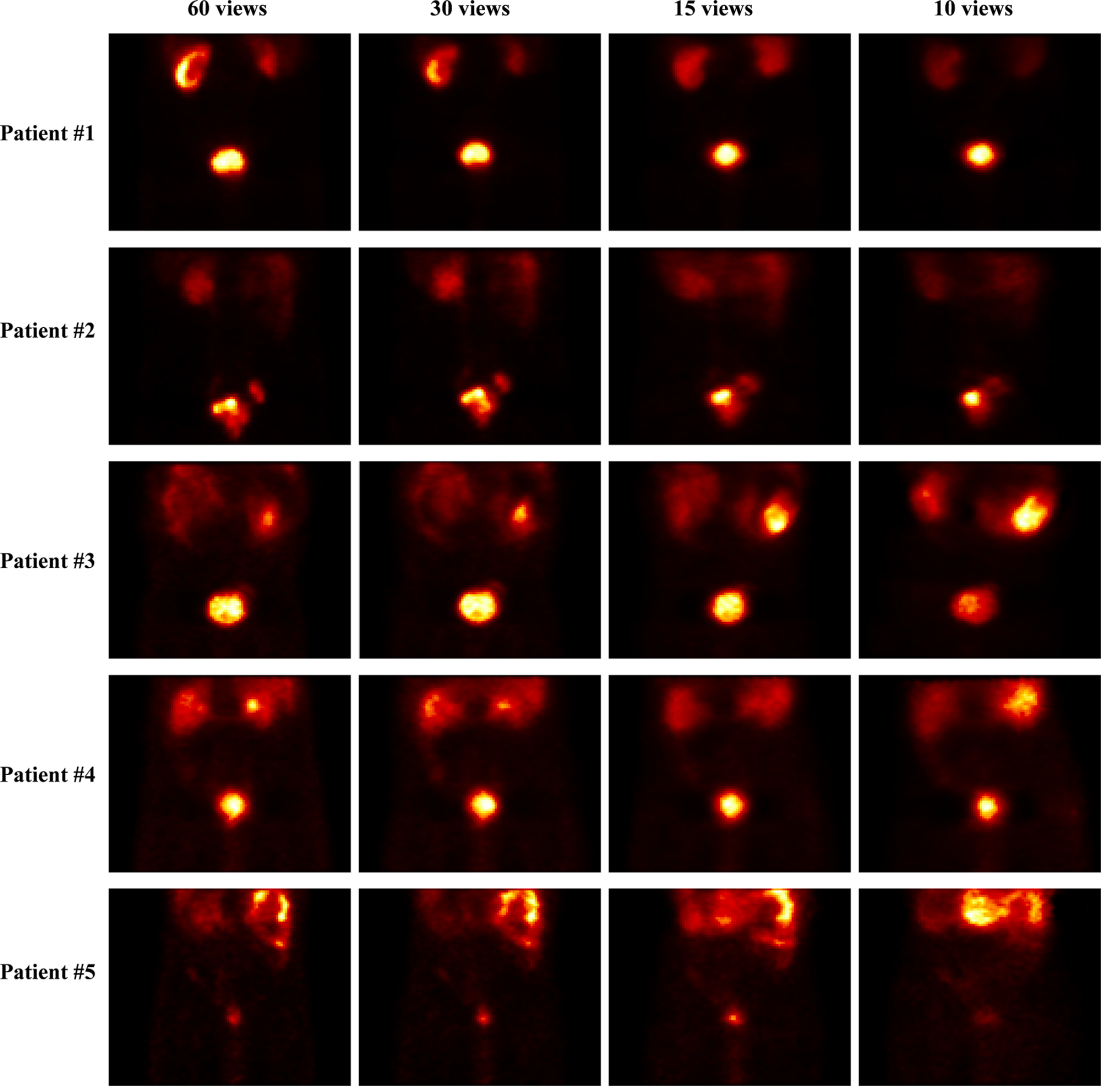
SPECT images with the OSEM algorithm at 60, 30, 15, and 10 views (patients number 1 to number 5). OSEM, ordered subset expectation maximization; SPECT, single photon emission computed tomography.

**Fig. 2:**
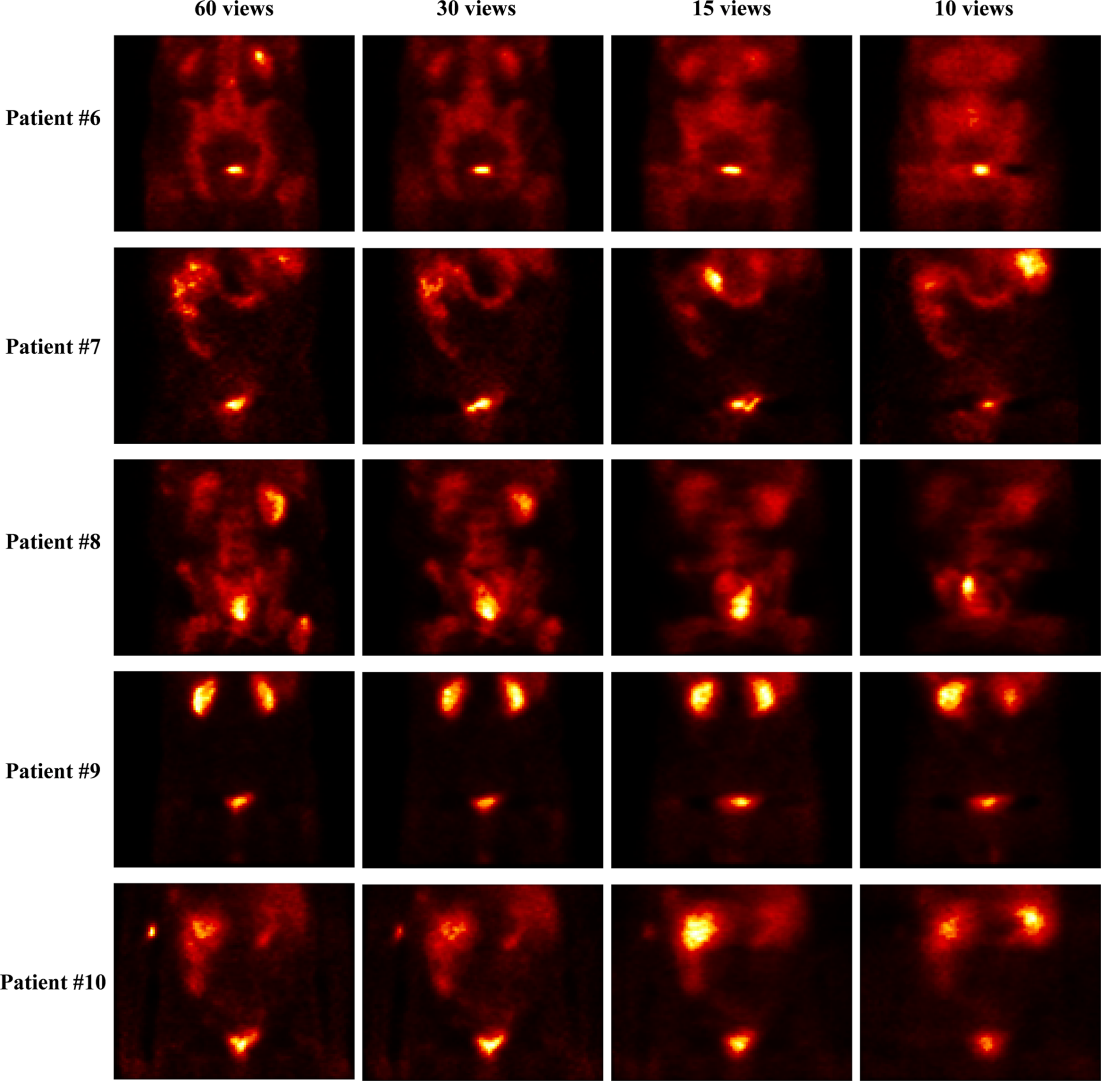
SPECT images with the OSEM algorithm at 60, 30, 15, and 10 views (patients number 6 to number 10). OSEM, ordered subset expectation maximization; SPECT, single photon emission computed tomography.

Initially, a comparison of the reconstructed data with only the OSEM algorithm from total views (60 views), 30, 15, and 10 views is presented in Figs. 1 and 2. As illustrated in the figures, when the number of views is reduced to 30, despite the appearance of some changes in image structure and the emergence of artifacts in all patients, the overall image structure, as well as the critical regions like the prostate area, containing valuable information, remains clear. This suggests that the diagnosis of abnormalities is still feasible. However, with the reduction to 15 views, the artifacts become more pronounced in the reduced images. In the 15-view images of all patients, it is evident that some information is lost, yet identifying other critical regions is still possible. In contrast, the situation with the 10-view reduced images is markedly different. The 10-view images exhibit severe artifacts, and the overall image structure is significantly compromised. Nevertheless, to accurately assess these reduced images, it is necessary to apply quantitative evaluation metrics.

**Fig. 3:**
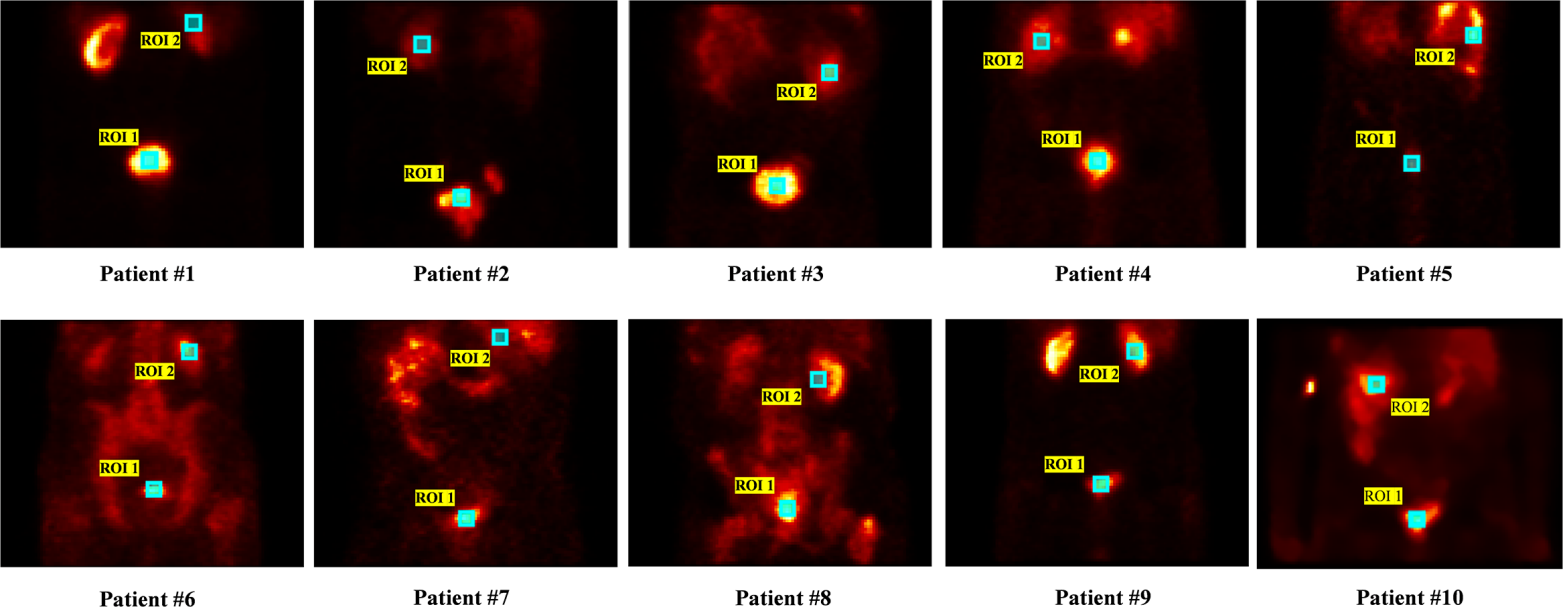
ROIs were drawn on the SPECT images of 10 patients: ROI1 (prostate lesion), ROI2 (physiologic reference-renal cortex). ROIs, region of Interest; SPECT, single photon emission computed tomography.

Figure 3 depicts the ROI maps selected for each image, where ROI 1 is the focal prostate lesion and ROI 2 is the renal cortex, and Table 2 reports the results of the MAPE and NRMSE metrics from Figs. 1 and 2. Based on the values reported in Table 2, as expected, the MAPE values in both ROIs for the 30-view images are reasonably below 31%. The MAPE value for the 30-view images was even reported to be as low as 0.66% for one of the patients. Considering that the goal of this imaging is to measure the radiopharmaceutical uptake in the prostate when the number of views is reduced to half of the initial amount (30 views), the MAPE value in the primary ROI1 is reported to be only around 7% for all patients on average. These values are noteworthy, given the significantly reduced number of views.

**Table 2:** The results of the mean absolute percentage error and normalized root mean square criteria for images with different numbers of views

The number of views	Patients	MAPE (ROI1)	MAPE (ROI2)	NMRSE
30	#1	15.27%	5.64%	0.0968
	#2	13.48%	4.97%	0.1777
	#3	1.81%	4.22%	0.1014
	#4	3.32%	5.07%	0.0635
	#5	3.78%	12.32%	0.1314
	#6	5.94%	18.10%	0.1684
	#7	2.15%	30.23%	0.1311
	#8	9.56%	0.84%	0.0926
	#9	13.64%	2.15%	0.1256
	#10	0.66%	12.22%	0.1017
15	#1	28.15%	16.71%	0.1118
	#2	22.57%	35.16%	0.2189
	#3	6.70%	32.14%	0.1651
	#4	38.74%	7.11%	0.0991
	#5	23.11%	25.38%	0.1722
	#6	10.09%	18.90%	0.2550
	#7	6.97%	51.90%	0.1526
	#8	11.42%	8.30%	0.0974
	#9	41.91%	28.05%	0.2192
	#10	10.94%	44.17%	0.1679
10	#1	85.48%	60.69%	0.4540
	#2	29.28%	69.02%	0.2872
	#3	32.31%	90.78%	0.3831
	#4	51.23%	37.71%	0.1861
	#5	61.44%	40.51%	0.2208
	#6	17.08%	35.75%	0.3099
	#7	53.59%	59.87%	0.2491
	#8	31.70%	15.74%	0.1470
	#9	58.26%	52.80%	0.2832
	#10	17.50%	45.66%	0.2471

MAPE, mean absolute percentage error; ROI, region of interest.

However, the error rate for the 10-view images, for one patient, the MAPE value even exceeds more than 85%, in the main region (ROI 1), and for all patients is around 50% on average. Given the visual assessment of the images, the presence of artifacts, the poor image structure in the 10-view images, and the high error rates, it can be concluded that reducing the number of views to 10 is not feasible. Among the reduced protocols, only 30 views were considered viable. Although the error rate of the 15-view study shows that it needs further study. As a result, in this study, the results of the 30- and 15-view will be investigated thoroughly.

Nevertheless, as mentioned in Sections ‘Introduction’ and ‘Ordered subset expectation maximization-nonlocal means algorithm’ regarding the source of noise in the reconstructed images, applying an optimization filter is essential.

**Fig. 4:**
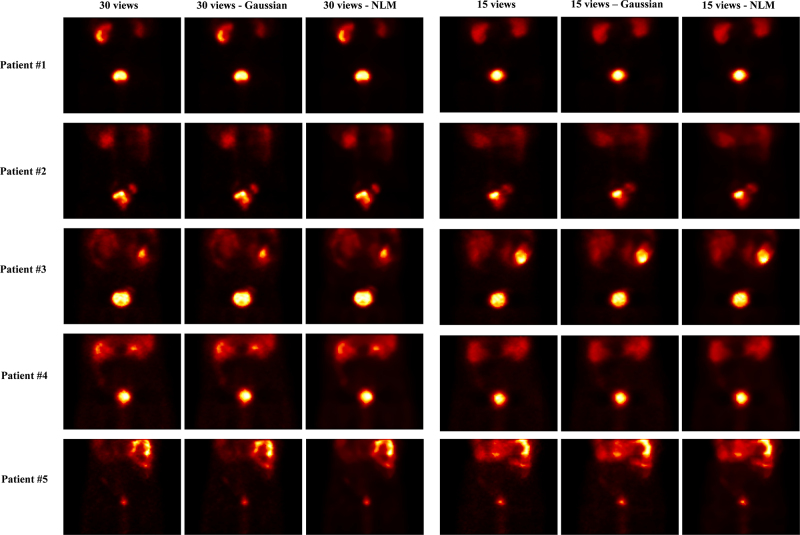
Comparison of OSEM, OSEM+Gaussian, and OSEM+NLM for 30 and 15 views (patients number 1 to number 5). NLM, nonlocal means; OSEM, ordered subset expectation maximization.

**Fig. 5:**
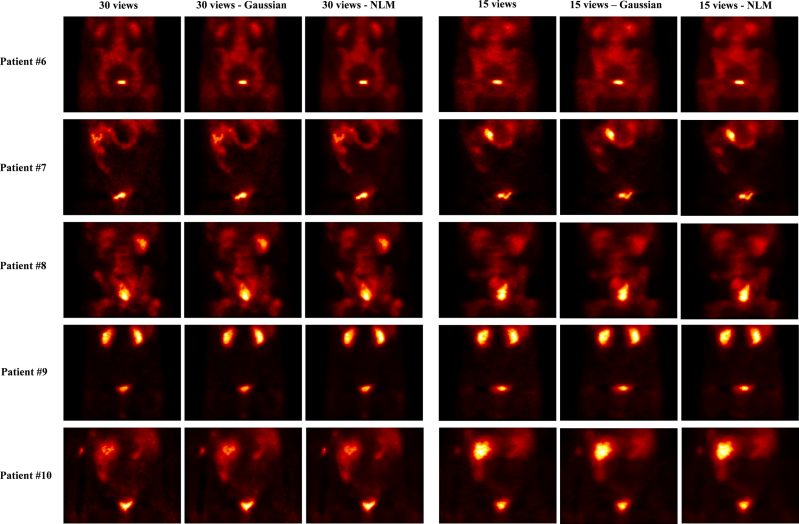
Comparison of OSEM, OSEM+Gaussian, and OSEM+NLM for 30 and 15 views (patients number 6 to number 10). NLM, nonlocal means; OSEM, ordered subset expectation maximization.

As shown in Figs. 4 and 5, the images are displayed in three categories: reconstructed with the OSEM algorithm without applying any filter, with a Gaussian filter, and with an NLM filter with 30 and 15 views. As is evident from the comparison of the images, the NLM filter has been more effective than the Gaussian filter in reducing noise while preserving image information. However, for a more precise evaluation, quantitative analysis is always required.

For quantitative evaluations, four metrics are utilized: MAPE, NRMSE, PSNR, and NB. Tables 3 and 4 present the evaluation metrics for the ten patients for 30 and 15 views, respectively, based on Figs. 4 and 5. By reviewing the results in these tables and comparing the values, it is clear that the NLM filter exhibits superior performance in optimizing the images.

**Table 3:** The results of the evaluation criteria for the single photon emission computed tomography images with 30 views

Patients	Methods	MAPE (ROI1)	MAPE (ROI2)	NRMSE	PSNR	NB (ROI1)
#1	No filter	15.27%	5.64%	0.0968	92.44	1.91
	Gaussian	14.79%	5.20%	0.0945	93.31	1.88
	NLM	**13.34%**	**5.08%**	**0.0778**	**94.00**	**1.83**
#2	No filter	13.48%	4.97%	0.1777	95.71	1.84
	Gaussian	12.81%	4.12%	0.1479	97.29	1.56
	NLM	**10.57%**	**3.67%**	**0.1078**	**100.04**	**1.50**
#3	No filter	1.81%	4.22%	0.1014	96.28	0.30
	Gaussian	1.68%	4.01%	0.0997	96.42	0.27
	NLM	**1.52%**	**3.29%**	**0.0909**	**97.22**	**0.23**
#4	No filter	3.32%	5.07%	0.0635	97.33	0.59
	Gaussian	3.06%	4.99%	0.0622	97.52	0.56
	NLM	**2.87%**	**4.88%**	**0.0566**	**98.34**	**0.46**
#5	No filter	3.78%	12.32%	0.1314	106.02	0.66
	Gaussian	2.38%	10.90%	0.1227	106.61	0.61
	NLM	**1.25%**	**7.99%**	**0.0969**	**108.67**	**0.54**
#6	No filter	5.94%	18.10%	0.1684	100.37	0.99
	Gaussian	3.69%	17.79%	0.1599	100.83	0.85
	NLM	**1.47%**	**16.80%**	**0.1323**	**102.47**	**0.81**
#7	No filter	2.15%	30.23%	0.1311	94.75	0.67
	Gaussian	0.89%	28.43%	0.1213	95.70	0.40
	NLM	**0.31%**	**25.32%**	**0.0946**	**96.68**	**0.35**
#8	No filter	9.56%	0.84%	0.0926	83.56	1.94
	Gaussian	8.88%	0.70%	0.0893	83.88	1.77
	NLM	**7.96%**	**0.53%**	**0.0787**	**84.98**	**1.58**
#9	No filter	13.64%	2.15%	0.1256	104.17	1.72
	Gaussian	11.72%	1.79%	0.1170	104.79	1.54
	NLM	**10.73%**	**0.93%**	**0.1030**	**105.89**	**1.39**
#10	No filter	0.66%	12.22%	0.1017	100.97	0.30
	Gaussian	0.64%	3.24%	0.0990	101.20	0.22
	NLM	**0.45%**	**2.94%**	**0.0925**	**101.79**	**0.09**

MAPE, mean absolute percentage error; NB, normalized bias; NLM, nonlocal means; NRMSE, normalized root mean square; PSNR, peak signal-to-noise ratio; ROI, region of interest. Values typeset in boldface mark the more desirable value.

**Table 4:** The results of the evaluation criteria for the single photon emission computed tomography images with 15 views

Patients	Methods	MAPE (ROI1)	MAPE (ROI2)	NRMSE	PSNR	NB (ROI1)
#1	No filter	28.15%	16.71%	0.1118	90.86	3.26
	Gaussian	27.78%	16.62%	0.1103	90.97	3.20
	NLM	**27.01%**	**16.13%**	**0.0974**	**92.10**	**3.12**
#2	No filter	22.56%	35.16%	0.2189	94.48	2.97
	Gaussian	21.94%	24.83%	0.2031	94.55	2.93
	NLM	**19.82%**	**33.63%**	**0.1887**	**94.80**	**2.88**
#3	No filter	6.70%	32.14%	0.1651	92.05	1.49
	Gaussian	6.14%	31.85%	0.1627	92.17	1.44
	NLM	**5.82%**	**30.47%**	**0.1467**	**93.07**	**1.36**
#4	No filter	38.74%	7.11%	0.0991	93.47	8.18
	Gaussian	36.66%	6.90%	0.0976	93.60	7.95
	NLM	**34.10%**	**5.98%**	**0.0867**	**94.63**	**7.63**
#5	No filter	23.11%	25.38%	0.1722	103.67	3.79
	Gaussian	22.02%	24.97%	0.1580	104.42	3.69
	NLM	**19.07%**	**23.83%**	**0.1229**	**106.60**	**3.53**
#6	No filter	10.09%	18.90%	0.2550	96.77	1.32
	Gaussian	7.43%	18.52%	0.2483	97.00	1.21
	NLM	**5.99%**	**17.41%**	**0.2229**	**97.94**	**1.10**
#7	No filter	6.97%	51.90%	0.1526	91.43	1.63
	Gaussian	6.36%	50.43%	0.1490	92.88	1.58
	NLM	**4.64%**	**48.64%**	**0.1371**	**94.16**	**1.55**
#8	No filter	11.42%	8.30%	0.0974	83.13	2.01
	Gaussian	11.04%	6.24%	0.0853	83.31	1.96
	NLM	**10.36%**	**5.17%**	**0.0930**	**84.53**	**1.80**
#9	No filter	41.91%	28.05%	0.2192	99.33	10.01
	Gaussian	39.22%	27.45%	0.2000	100.13	9.32
	NLM	**37.70%**	**26.83%**	**0.1553**	**102.33**	**9.09**
#10	No filter	10.94%	44.17%	0.1679	97.23	1.43
	Gaussian	10.19%	43.66%	0.1645	97.99	1.39
	NLM	**9.24%**	**43.54%**	**0.1598**	**98.41**	**1.27**

MAPE, mean absolute percentage error; NB, normalized bias; NLM, nonlocal means; NRMSE, normalized root mean square; PSNR, peak signal-to-noise ratio; ROI, region of interest. Values typeset in boldface mark the more desirable value.

To evaluate the amount of improvement for all metrics, it is measured based on the percentage change (PC) as follows:


$$\begin{array}{l} PC=\frac{x_{2}-x_{1}}{x_{1}}\times 100 \end{array}$$ (13)

where *x*_*1*_ is the initial value and *x*_*2*_ is the final value.

Based on the evaluation of the data obtained from the 30-view images of all patients, regarding the PC, the values are as follows: according to the MAPE metric, in ROI 1, the NLM-filtered images show up to 4.47% improvement compared to the unfiltered images and up to 2.24% improvement compared to the Gaussian-filtered images. In ROI 2, the improvement is up to 5.09% compared to the unfiltered images and 3.11% compared to the Gaussian-filtered images. The optimization in PSNR, NRMSE, and NB metrics for the NLM filter, compared to the unfiltered images, is up to 4.52, 39.33, and 18.55%, respectively, and compared to the Gaussian-filtered images, is up to 2.82, 27.11, and 13.01%, respectively.

For the data obtained from the 15-view images of all patients, the PC values in image quality based on the MAPE metric are as follows: in ROI1, the NLM-filtered images show up to a 5.90% improvement compared to the unfiltered images and 2.24% compared to the Gaussian-filtered images. In ROI 2, the improvement is up to 3.26% compared to the unfiltered images and 2.27% compared to the Gaussian-filtered images. The optimization in PSNR, NRMSE, and NB metrics for the NLM filter, compared to the unfiltered images, is up to 3.02, 29.15, and 9.19% respectively, and compared to the Gaussian-filtered images, 2.82, 22.35, and 4.02%, respectively.

In addition to the quantitative image quality metrics, a detailed visual assessment was conducted by two experienced nuclear medicine physicians. For each patient, comparisons were made between images reconstructed with 30 projections filtered using NLM vs. Gaussian filters. Both experts independently scored the images based on diagnostic clarity and visual noise suppression. The interobserver agreement was calculated using the intraclass correlation coefficient (ICC), which demonstrated good to excellent reliability. The scoring outcomes consistently indicated that the NLM filter provided superior visual quality compared to the Gaussian filter in the 30-view scenario. This finding further confirms the effectiveness of NLM filtering in enhancing image interpretability, especially in sparse-view conditions. Table 5 demonstrates the evaluations obtained from physicians.

**Table 5:** Subjective image quality scores by physicians for 30 views

Patients	Filters	Physician 1	Physician 2
#1	Gaussian	4	4
	NLM	5	5
#2	Gaussian	4	4
	NLM	5	5
#3	Gaussian	4	4
	NLM	5	5
#4	Gaussian	5	5
	NLM	5	5
#5	Gaussian	4	4
	NLM	5	5
#6	Gaussian	4	4
	NLM	5	5
#7	Gaussian	4	4
	NLM	5	5
#8	Gaussian	3	3
	NLM	4	4
#9	Gaussian	5	5
	NLM	5	5
#10	Gaussian	4	4
	NLM	5	5

NLM, nonlocal means.

To assess the consistency between the two expert readers in visual scoring of image quality, the ICC is calculated. The ICC for the 30-view images was 0.83, indicating good agreement. This supports the reliability of visual reports. The ICC value has been obtained based on the following formula:


$$\begin{array}{l} ICC\left(1,2\right)=\frac{\left(MSB-MSE\right)}{\left(MSB+\left(K-1\right)\times MSE\right)} \end{array}$$ (14)

where MSB is the mean square between subjects that measures variability between subjects (patients), MSE is the mean square error that measures variability not explained by the model (residual error), and *K* is the number of raters (e.g. two physicians).

## Discussion

The SPECT scanner is a critical medical device for the diagnosis, assessment, and treatment of a wide range of diseases and abnormalities. However, the lengthy preparation time, particularly for patient preparation and image acquisition are significant concern. In this study, we aimed to reduce the number of views in SPECT imaging using a limited-angle approach. In this study, the total number of views acquired from the ^99m^Tc-PSMA images was 60, which was reduced to 30, 15, and 10 views. The resulting images were reconstructed using the iterative OSEM algorithm, and an evaluation based on the MAPE and NRMSE metrics was conducted. The analysis revealed that images with 30 views produced precise results. Considering that the number of views was halved, these results are highly significant. The error rate of the 15-view images was higher than that of the 30-view images, but the fact that the number of views was reduced to a quarter of the original still makes these findings important. As a result, it concluded that further investigation of the 15-view images is needed. However, the images from 10 views suffered from severe artifacts, with the overall image structure wholly lost, and the reported error rate was extremely high. Based on the evaluations of these three viewpoints, it is reliable to conclude that reducing the number of views to 10 is not feasible. As a result, further analysis of the 10-view images was omitted from the study.

After image reconstruction, the filtering stage to remove noise is a critical step. One of the most common filters used is the Gaussian filter; however, it has been shown that the NLM filter performs better in noise reduction. As a result, the reduced images from 30 to 15 views were optimized using both Gaussian and NLM filters. The visual assessment demonstrated that the NLM filter outperformed the Gaussian filter even in the limited-angle setting. Numerical evaluation metrics also confirmed the superior performance of the NLM filter. Based on the PC value, an improvement of up to 39.33% was observed in NLM-filtered images for 30 views and up to 29.15% for 15 views.

However, the study concluded that angular down-sampling in tomographic imaging operates as a sampling-rate reduction along the projection angle dimension. When the angular increment becomes too large, object spatial frequencies beyond the corresponding angular Nyquist limit are folded back (‘aliasing’), producing structured streaks and edge-like artifacts. In this study, decimating from the native 6 ° step to 12 ° (30 views) preserved essential frequency content for ^99m^Tc-PSMA pelvic imaging, whereas more aggressive decimations to 24 ° (15 views) and 36 ° (10 views) produced fold-over artifacts that are visually evident in Figs. 1, 2, 4, and 5. Because NLM is an edge-preserving denoiser, it is not designed to correct aliasing and may even enhance boundaries of aliased regions if antialiasing is not performed upstream. Consistent with these sampling-theoretic considerations, therefore, this study does not endorse 15- or 10-view reconstructions for clinical use in this dataset and limits the feasibility claim to 30 views.

It is worth noting that in this study, the protocol with 30 projections and more time per projection was used because this method is easily implementable in a clinical setting and aids in better management of patient movement. Patient movement can corrupt some projections, and with fewer projections, identifying and correcting these damaged projections is easier. This is particularly important for patients who have difficulty remaining still. Research such as Takahashi *et al*. [30] has shown that even with a few projections (such as 30) with longer acquisition time, using appropriate reconstruction algorithms, it is possible to obtain images of acceptable quality in comparison to more projections (60 and 120) with less imaging time.

In addition to the quantitative evaluation metrics (e.g. MAPE, NRMSE, PSNR, and NB), a qualitative assessment was performed by two experienced nuclear medicine physicians who independently scored the reconstructed images obtained using Gaussian and NLM filters in the 30-view settings. Their scoring was based on clinical usability, lesion conspicuity, and overall image clarity. To assess the consistency between the two observers, the ICC was calculated. The ICC for the 30-view images was 0.83, indicating good agreement. These findings support the robustness and reproducibility of expert-based image evaluation. Although the current study does not include direct clinical validation, such as lesion detection accuracy or diagnostic outcomes, the strong agreement in visual assessments provides a meaningful proxy for clinical relevance. Future research will focus on further validation using phantoms to analyze spatial resolution degradation, point spread function, and modulation transfer function, which are critical for fully characterizing the imaging performance under sparse-view acquisition protocols.

Sparse-view acquisition itself is an established, routinely configurable option on commercial systems; this study’s contribution is the PSMA-SPECT context and the evaluation of an NLM-based postreconstruction filter. While limited angular sampling has been a well-established practice in cardiac SPECT since the early 1980s [13,14], its application in ^99m^Tc-PSMA SPECT for prostate cancer is novel. This study demonstrates that 30-view acquisition can yield diagnostically viable images when combined with OSEM and edge-preserving denoising. By contrast, 15-view reconstructions in the setting showed aliasing consistent with the violation of angular Nyquist sampling and are not recommended for clinical use without additional antialias measures and validation. This study also provides detailed parameterization for both reconstruction and denoising, enabling reproducibility and further optimization.

However, the small sample size (*n* = 10) and retrospective design limit statistical power. Future studies should enroll larger scanning conditions and incorporate advanced AI-based denoising to further enhance image quality.

## Conclusion

In this study, a sparse-view reconstruction approach was applied to ^99m^Tc-PSMA SPECT using OSEM followed by postprocessing with Gaussian and NLM filters. Quantitative and visual assessments demonstrated that 30-view reconstructions yielded accurate and clinically acceptable results. 15-view reconstructions, however, displayed sampling-related artifacts consistent with angular undersampling and are not endorsed for clinical use in this study’s dataset. The NLM filter consistently outperformed Gaussian filtering in suppressing noise, but, as an edge-preserving denoiser, it does not correct aliasing. These findings support 30-view acquisition as a practical time-saving protocol while underscoring the need for explicit antialiasing and further validation before considering more aggressive decimation. Complementary visual assessments conducted by two nuclear medicine experts confirmed these findings, with high interobserver agreement (ICC = 0.83 for 30 views), reinforcing the clinical relevance of the reconstructed images. These results highlight the feasibility of reducing acquisition views without significantly compromising diagnostic quality.

## Acknowledgements

The authors would like to express their heartfelt gratitude to the Nuclear Medicine Research Center of Qaem Hospital for their invaluable support and resources throughout this study. A special thanks goes to Dr Atene Aghaee for their exceptional guidance, encouragement, and insightful contributions, which were instrumental in the completion of this work.

This research received no specific grant from any funding agency in the public, commercial, or not-for-profit sectors.

This study was conducted following the principles of the Declaration of Helsinki. Ethical approval was obtained from the Research Ethics Committee of Ghaem Hospital, Mashhad, Iran, and the Vice-Chancellor for Research of Mashhad University of Medical Sciences under the approval number of IR.MUMS.MEDICAL.REC.1401.268. The research protocol was reviewed and approved at both institutional levels. Informed consent was also obtained from all individual participants included in the study.

### Conflicts of interest

There are no conflicts of interest.
